# Highly thermostable mixed lanthanide organic frameworks with high quantum yield for warm white light-emitting diodes

**DOI:** 10.3389/fchem.2023.1204646

**Published:** 2023-05-22

**Authors:** Yanqiong Shen, Xianyong Pan, Yaru Zhao, Qingchuan Gu, Qipeng Li

**Affiliations:** ^1^ College of Chemistry and Chemical Engineering, Zhaotong University, Zhaotong, China; ^2^ School of Physics and Information Engineering, Zhaotong University, Zhaotong, China

**Keywords:** high thermostability, high quantum yield, mixed lanthanide organic frameworks, warm white light-emitting diode, colour rendering index

## Abstract

A mixed lanthanide organic framework was prepared *via* hydrothermal methods using *m*-phthalic acid (*m*-H_2_BDC), 1,10-phenanthroline (1,10-Phen), and Ln^3+^ ions, formulated as [HNMe_2_][Eu_0.095_Tb_1.905_(*m*-BDC)_3_(phen)_2_] (**ZTU-6**). The structure and stability of **ZTU-6** were characterised by X-ray diffraction (XRD) and thermogravimetric analysis (TGA), which revealed a three-dimensional *pcu* topology with high thermal stability. Fluorescence tests showed that **ZTU-6** emitted orange light with a high quantum yield of 79.15%, and it can be effectively encapsulated in a light-emitting diode (LED) device emitting orange light. In addition, **ZTU-6** was found to be compatible with BaMgAl_10_O_17_:Eu^2+^ (BAM) blue powder and [(Sr,Ba)_2_SiO_4_:Eu^2+^] silicate yellow and green powder to create a warm white LED with a high colour rendering index (CRI) of 93.4, a correlated colour temperature (CCT) of 3908 K, and CIE coordinates of (0.38, 036).

## 1 Introduction

Metal–organic frameworks (MOFs) are a new class of organic–inorganic hybrid materials formed by the self-assembly of metal ions or metal clusters and organic ligands through coordination bonds (Maurin et al., 2017). MOFs are organic and inorganic with adjustable pore structures and sizes and have potential applications in the fields of magnetic materials, fluorescence sensing, gas adsorption and separation, luminescent materials, and so forth (Wycho Waniec et al., 2022; Bai et al., 2016; Wu et al., 2021; Huang et al., 2023; Zhang et al., 2020). Lanthanide organic frameworks are synthesised using lanthanide ions as the central metal owing to their unique 4f electron layer structure that undergoes various transitions, leading to fluorescence emissions of different colours (Cui et al., 2018). In lanthanide organic frameworks, the organic ligands mainly connect the lanthanide ions, adjust the size of the material, and effectively transfer energy to the lanthanide ions, thereby improving the luminous intensity of the lanthanide ions through the antenna effect (Tang et al., 2020; Tsai et al., 2021). In addition, white-light emission from lanthanide organic frameworks can be realised by adjusting the proportion of lanthanide ions, material temperature, and excitation wavelength (Tang et al., 2020; Tsai et al., 2021).

In recent years, with the implementation of the strategy of peak carbon emissions and carbon neutrality, light-emitting diodes (LEDs) have gradually replaced fragile, low efficiency, and high energy consumption incandescent lamps as well as fluorescent and high-pressure mercury lamps that are fragile, toxic, and pollute the environment. LEDs have the advantages of high efficiency, energy saving, long life, no pollution, small size, and light weight and have become the fourth generation of light sources (Wu et al., 2016; Wei et al., 2019). Currently, common white LED (WLED) devices are primarily packaged using the following three methods (Cho et al., 2017; Yuan et al., 2021): I) white light through the combination of red, green, and blue LED multichips; II) ultraviolet LED excitation phosphor-emitting three-colour synthetic white light; III) blue light LED excitation yellow phosphor to achieve white light emission. Although many WLED devices have been developed based on these three methods, each with advantages and disadvantages, there is still an urgent need to create high-quality and high-stability WLED devices with low correlated colour temperature (CCT), high colour rendering index (CRI), and high luminous efficacy that can be used in various challenging environments.

In this study, a mixed lanthanide organic framework (**ZTU-6**) with high thermal stability and high quantum yield was prepared using hydrothermal methods. **ZTU-6** can be encapsulated in warm WLED devices with excellent CRI and CCT, providing design ideas and theoretical references for the development of new WLED devices.

## 2 Materials and methods

### 2.1 Materials

All chemical reagents were purchased commercially and used without further purification. X-ray powder diffraction (XRD) was performed on **ZTU-6** using a Bruker D8 Advance diffractometer (Cu-K*α* radiation, *λ* = 0.154 nm). The thermal stability of **ZTU-6** was tested using a thermogravimetric analyser (Mettler Toledo, Switzerland). Luminescence tests were performed using an Edinburgh FLS980. The LED fluorescence performance of **ZTU-6** was measured using a Hangzhou HAAS-2000 photoelectric colour-integrated tester.

### 2.2 Preparation of ZTU-6

Based on previous literature (Zhang et al., 2011), *m*-H_2_BDC (167 mg, 1 mmol), 1,10-Phen (180 mg, 1 mmol), a certain amount of Eu(NO_3_)_3_·6H_2_O, and Tb(NO_3_)_3_·6H_2_O were dissolved in 3 mL *N*, *N*′-dimethylformamide (DMF) and 3 mL water in a 25 mL polytetrafluoroethylene reactor and then heated in an oven (120°C) for 72 h and subsequently cooled to room temperature. The prepared samples were washed three times with fresh DMF and acetone and dried at room temperature to obtain the crystal material **ZTU-6** (yield 41% based on the *m*-H_2_BDC ligand). Elemental analysis results (%) of C_50_H_35_Eu_0.095_Tb_1.905_N_5_O_12_ (1215.04): theoretical values C, 49.42; H, 2.90; N, 5.76; experimental values C, 49.15; H, 2.599; N, 5.64. The ratio of terbium to europium was 24.07:1.20 in **ZTU-6**, which was determined by inductively coupled plasma atomic emission spectrometry (ICP-AES).

### 2.3 Encapsulation and performance of LED devices

The **ZTU-6** crystal was fully ground, mixed with AB silica gel in a mass ratio of 1:1, and encapsulated on a commercial 365 nm UV LED chip to form the LED device. The LED device was heated at 150°C for 2 h, and its luminescence properties were tested using a HAAS-2000 instrument at room temperature. In addition, **ZTU-6** was combined with BaMgAl_10_O_17_:Eu^2+^ blue powder (BAM) and silicate yellow–green powder [(Sr,Ba)_2_SiO_4_:Eu^2+^] at a mass ratio of 3:1:6 and was encapsulated on a commercial 395 nm LED chip to form a warm WLED device (Li et al., 2020).

## 3 Results and discussion

### 3.1 Structural characterisation

**FIGURE 1:**
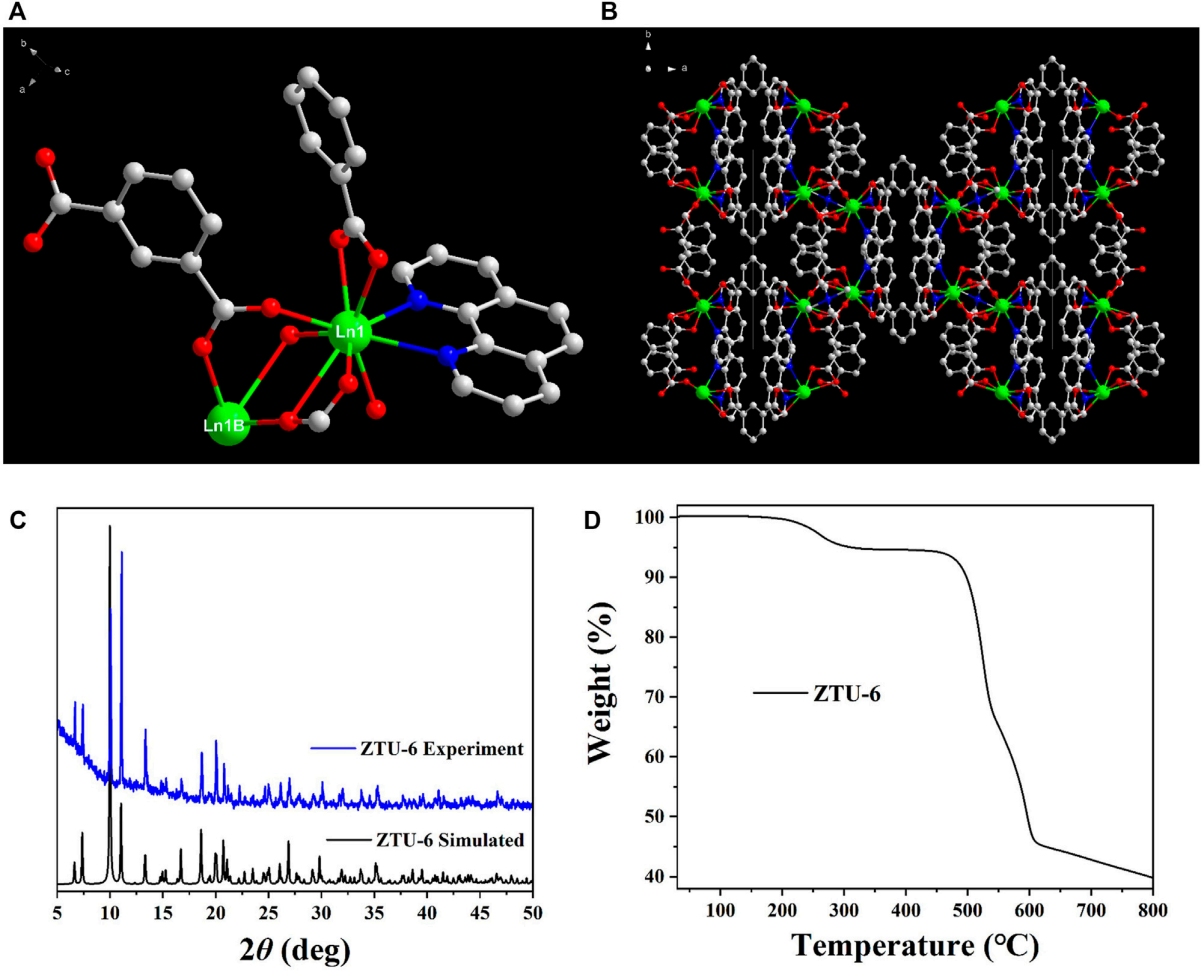
**(A)** Coordination environment of the central Ln^3+^ ions in **ZTU-6**, **(B)** 3D frameworks of **ZTU-6**, **(C)** XRD curves of **ZTU-6**, and **(D)** TGA curves of **ZTU-6**.

*m*-H_2_BDC, 1,10-Phen, and Ln^3+^ ions were used as examples of mixed lanthanide organic frameworks (**ZTU-6**) under hydrothermal conditions. The purity of **ZTU-6** was assessed by analysing its XRD pattern obtained using a Bruker D8 Advance instrument. The results shown in Figure 1C demonstrate that the experimental XRD peaks of **ZTU-6** corresponded to the simulated XRD peaks, confirming the successful preparation of **ZTU-6** in its pure phase (Zhang et al., 2011).

The asymmetric unit of **ZTU-6** has one Ln^3+^ ion, an *m*-BDC^2−^ ligand, one phenanthroline, and half a dimethylamine molecule. The central Ln^3+^ ions in **ZTU-6** are nine-coordinated with the seven oxygen atoms from five *m*-BDC^2−^ ligands and the two nitrogen atoms from 1,10-Phen (Figure 1A). Each Ln^3+^ ion connects to oxygen atoms, forming four bridging structures and two chelate structures with binuclear [Ln_2_(COO)_6_] secondary structure units (SBUs) (Zhang et al., 2011). In the structure, the *m*-BDC^2−^ ligand adopts two coordination modes. The first *m*-BDC^2−^ ligand adopts the (*k* ^2^-*μ*_2_)-(*k* ^1^-*k* ^1^)-*μ*_4_ mode, which connects the [Ln_2_(COO)_6_] SBU, forming a two-dimensional structure along the A-axis. The second *m*-BDC^2−^ ligand adopts the *k* ^2^-(*k* ^1^-*k* ^1^)-*μ*_3_ mode, which connects the adjacent two-dimensional layers (Zhang et al., 2011), forming a three-dimensional frame structure with the typical *pcu* topology (Figure 1B; Supplementary Figure S1).

The thermal stability of **ZTU-6** was tested under nitrogen at a heating rate of 10°C/min in the range of 20°C–800°C; weight loss of guest dimethylamine molecules occurred between 20°C and 300°C. The frameworks began to decompose after 550°C (Figure 1D; Supplementary Figure S2), indicating the high thermal stability of **ZTU-6**.

### 3.2 Analysis of the LED performance

**FIGURE 2:**
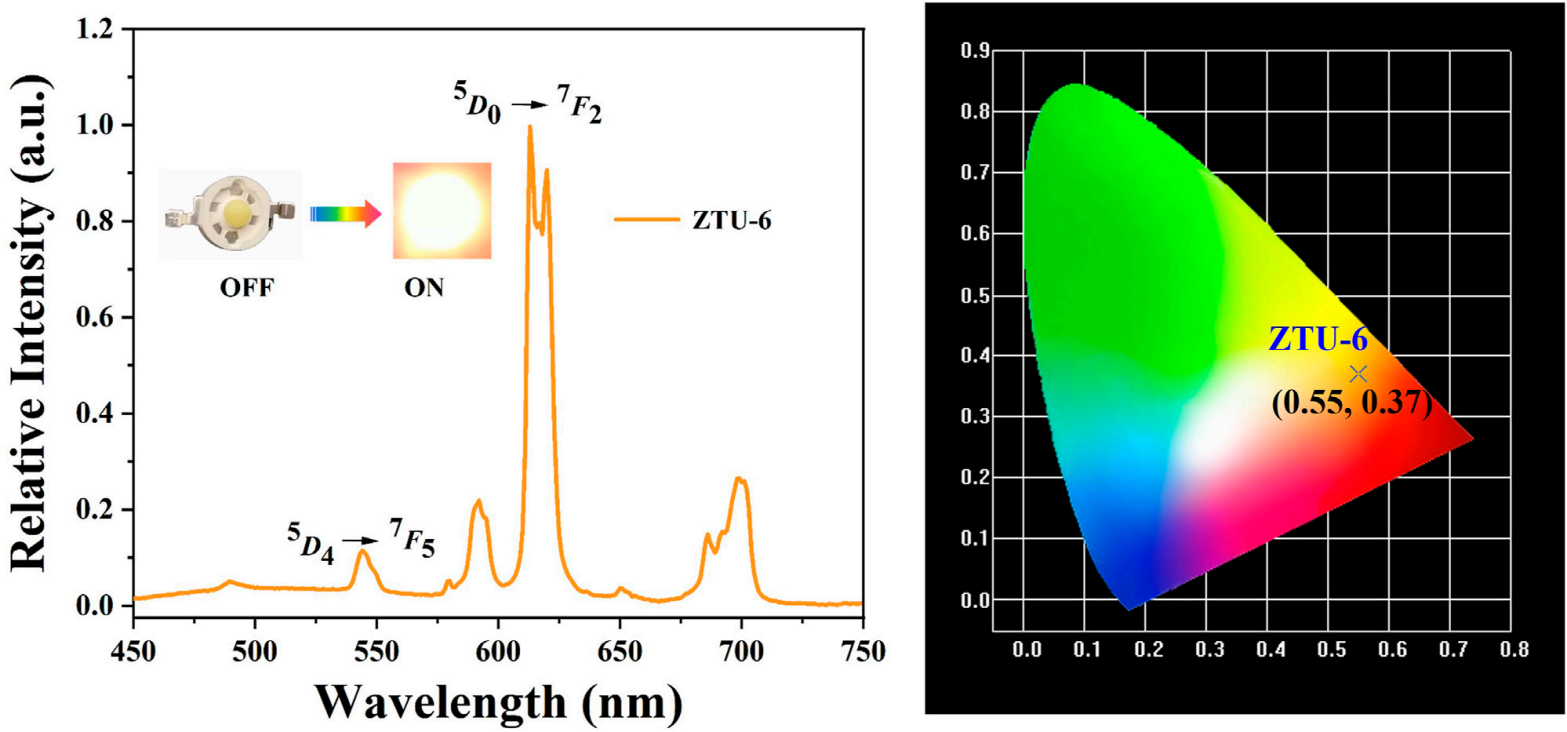
Emission spectroscopy and CIE coordinates of the LED device with **ZTU-6**.

Under excitation at λ_ex_ = 365 nm, **ZTU-6** emits a bright orange–yellow light with the characteristic emission (^5^ *D*_4_→^7^ *F*_5_) of Tb^3+^ ions at 544 nm and the characteristic emission (^5^ *D*_0_→^7^ *F*_2_) of Eu^3+^ ions at 613 nm (Figure 2), which indicates that the Tb^3+^ ions and Eu^3+^ ions simultaneously enter the framework at a ratio of 24.07:1.20 (Zhang et al., 2011; Li et al., 2020). Although the content of Eu^3+^ ions in **ZTU-6** is very low, the emission intensity is still dominant, indicating that Eu^3+^ and Tb^3+^ ions are in the same framework and that Eu^3+^ ions can be sensitised by Tb^3+^ ions (Zhang et al., 2011; Li et al., 2020). Furthermore, the quantum yield and fluorescence lifetime of **ZTU-6** were 79.15% and 1.09 m, respectively. The **ZTU-6** crystal material was fully mixed with AB silica gel in a 1:1 mass ratio and then encapsulated on a commercial 365 nm UV LED chip to obtain orange-light LED devices. At 20 mA, its CIE coordinates are (0.55, 0.37) (Figure 2), and its CCT is 1572 K.

**FIGURE 3:**
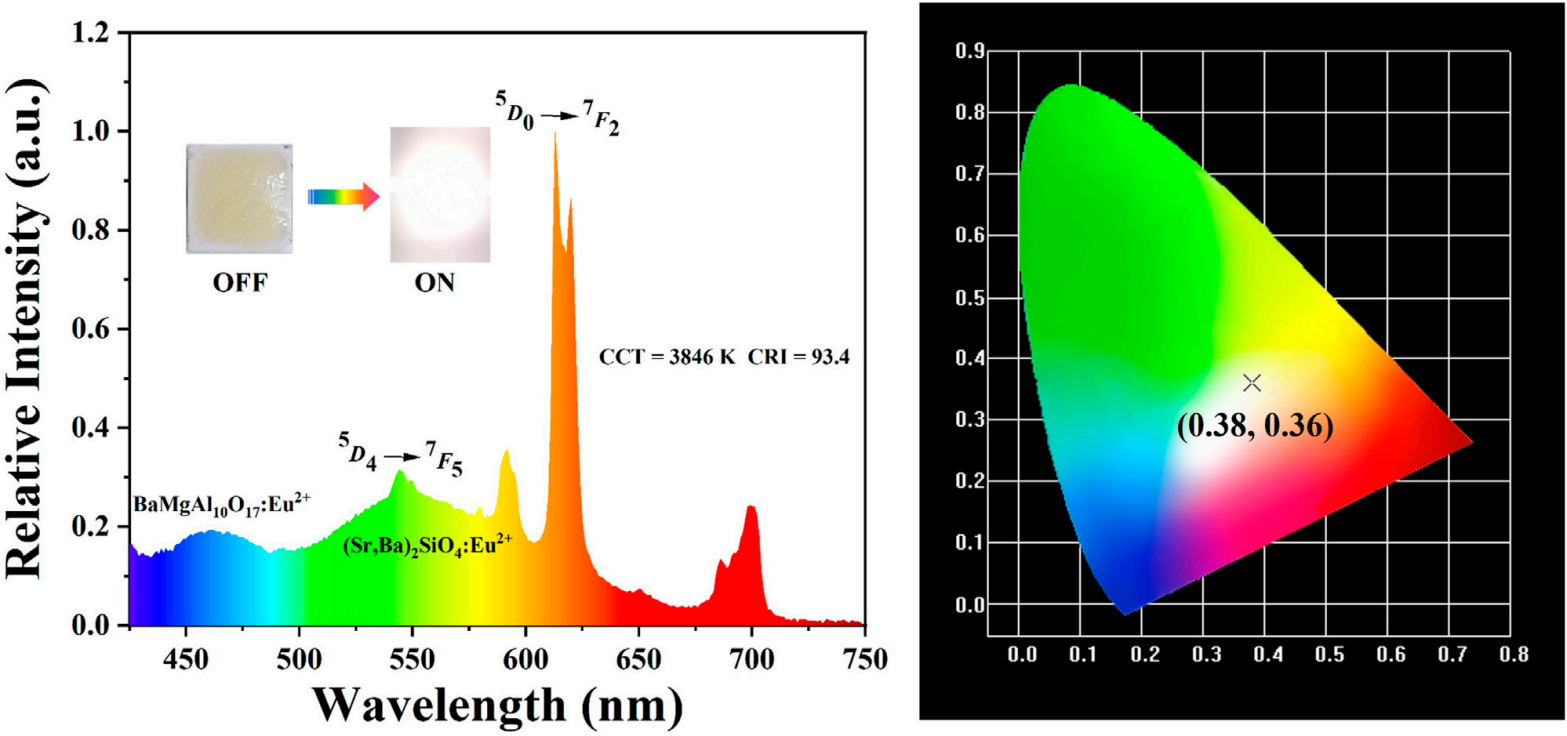
Emission spectrum and CIE coordinates of the warm WLED device fabricated using **ZTU-6**, BAM blue powder, and silicate yellow–green powder.

A warm WLED device was created by mixing **ZTU-6** with BAM blue powder and silicate yellow–green powder at a mass ratio of 3:1:6 and then encapsulating it on a commercial 395 nm LED chip. The warm WLED device emitted blue light at 460 nm from the BAM blue powder, the characteristic emission of Tb^3+^ ions at 544 nm, the characteristic emission of Eu^3+^ ions at 613 nm, and a yellow region from the commercial silicate yellow–green powder (Figure 3). At 20 mA, the device emitted warm white light with CIE chromaticity coordinates of (0.38,0.36), a CRI of 93.4, and a CCT of 3908 K, demonstrating excellent CRI and CCT and providing design ideas and theoretical references for developing new WLED devices (Wang et al., 2019; Gutiérrez et al., 2020; Karmakar and Li, 2022; Zhang et al., 2022).

## 4 Conclusion

A mixed lanthanide organic framework (**ZTU-6)** was successfully prepared using hydrothermal methods, and its structure was characterised. **ZTU-6** displays a 3-dimensional *pcu* topology with high thermal stability. In addition, **ZTU-6** emits orange light with a high quantum yield of 79.15%, and can be encapsulated into a warm-white-light LED device with excellent CRI and CCT obtained upon adding a commercial powder. The results provide design ideas and theoretical references for the development of new WLEDs.

## Data Availability

The original contributions presented in the study are included in the article/Supplementary Material; further inquiries can be directed to the corresponding authors.
