# Supplementary material for: Highly thermostable mixed lanthanide organic frameworks with high quantum yield for warm white light-emitting diodes
Source: Front Chem. 2023 May 22;11:1204646. doi: 10.3389/fchem.2023.1204646 (PMC10239934; doi:10.3389/fchem.2023.1204646)
Supplement: Supplementary file 1 [file DataSheet1.docx]

Supplementary Material

Highly Thermostable Mixed Lanthanide Organic Frameworks with High Quantum Yield for Warm White Light Emitting Diodes

Yanqiong Shen ^1^, Xianyong Pan ^1^, Yaru Zhao ^1^, Qingchuan Gu^2*^ and Qipeng Li ^1*^

^1^ College of Chemistry and Chemical Engineering, Zhaotong University, Zhaotong, 657000, P. R. China

^2^ Center of Information Technology and Education, Zhaotong University, Zhaotong, 657000, P. R. China

***Correspondence:** 53472864@qq.com and qpli@ztu.edu.cn

Preparation of the ZTU-6-Eu and ZTU-6-Tb

*m*-H_2_BDC (167 mg, 1 mmol), 1,10-Phen (180 mg, 1 mmol), a certain amount of Eu(NO_3_)_3_·6H_2_O (220 mg, 0.5 mmol) were dissolved in 3 mL *N*, *N*'-dimethylformamide (DMF) and 3 mL water in the 25 mL polytetrafluoroethylene reactor, which were heated in an oven (120 ℃) for 72 hours and cooled to room temperature. The prepared samples were washed three times with fresh DMF and acetone and dried at room temperature, to obtained the crystal material of **ZTU-6-Eu** (yield 48%, based on the *m*-H_2_BDC ligand). Whose elemental analysis result (%) C_50_H_35_Eu_2_N_5_O_12_ (1201.77): Theoretical value C，49.97；H，2.94；N，5.83; Experimental value 49.11；H，2.507；N，5.58.

Following the same procedure as described above, the Eu(NO_3_)_3_·6H_2_O change to Tb(NO_3_)_3_·6H_2_O (220 mg, 0.5 mmol), obtained the crystal material of **ZTU-6-Tb** (yield 53%, based on the *m*-H_2_BDC ligand), whose elemental analysis result (%) C_50_H_35_Tb_2_N_5_O_12_(1215.69): Theoretical value C, 49.40; H, 2.90; N, 5.76; Experimental value: C, 48.93; H, 2.597; N, 5.62.

#
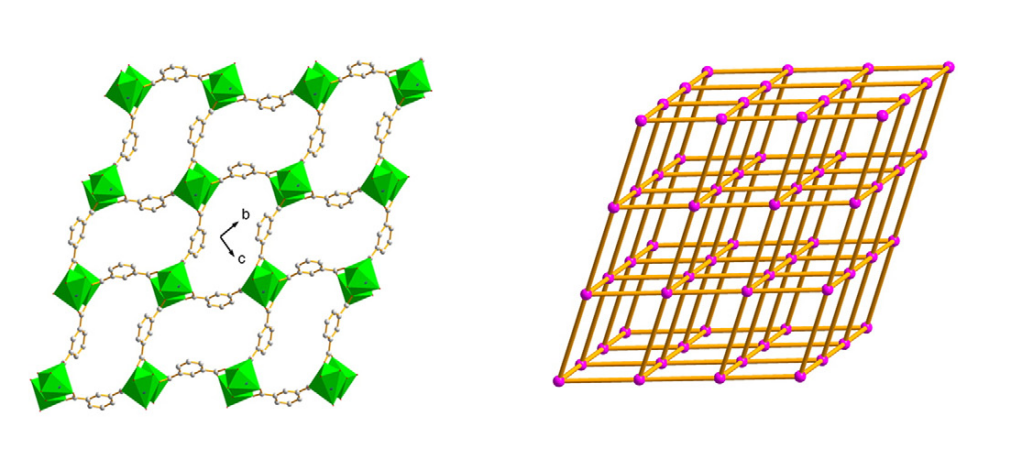


Figure S1 Structural and topological maps of **ZTU-6**


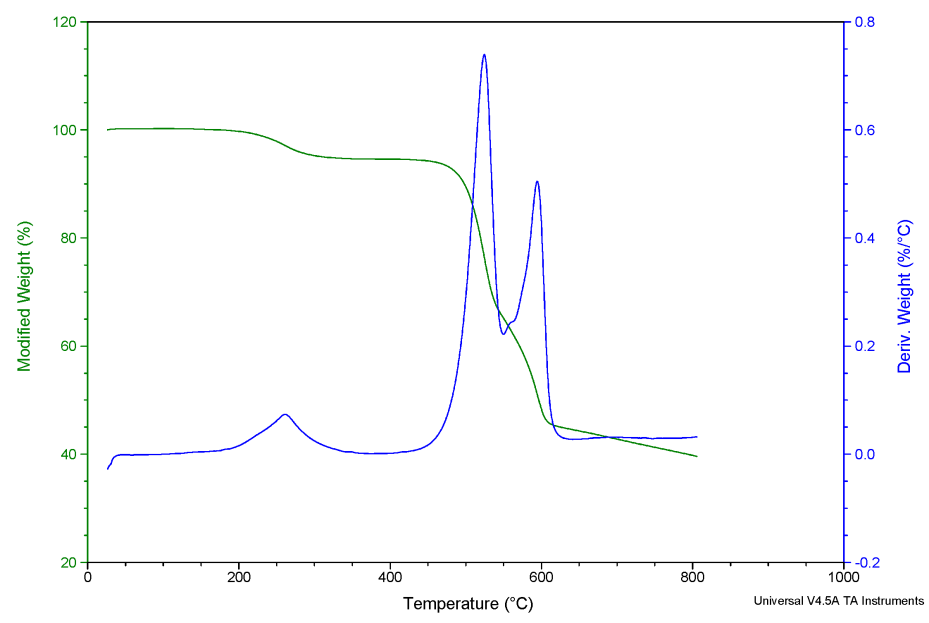


Figure S2 The TGA and DSC curves of **ZTU-6**

At λ_ex_ = 365 nm, **ZTU-6-Eu** shows a bright emitting red light with emission spectrum consists of several emission peaks in the wavelength region of 575-725 nm, which associated with the ^5^*D*_0_→^7^*F*_J_ (*J* = 1-4) and the strongest emission peak at 613 nm attributed to the transition of ^5^*D*_0_→^7^*F*_2_ (Figure S3)_._


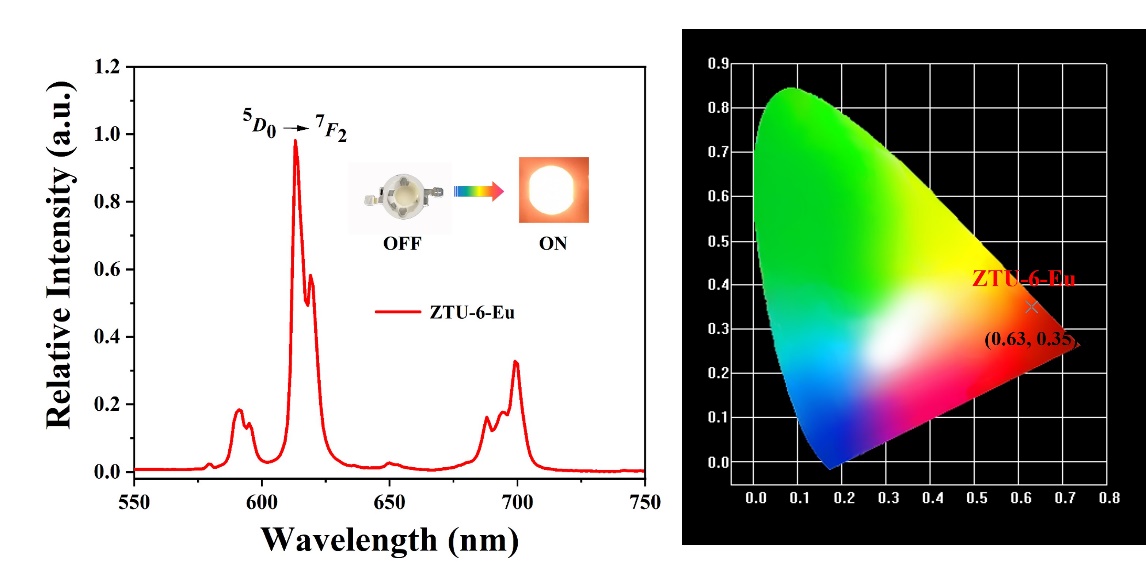


Figure S3 Emission spectroscopy and the CIE of the LED device with the **ZTU-6-Eu**.

Under the λ_ex_ = 365 nm, **ZTU-6-Tb** shows bright green light emission with the typical emission peaks at 490 nm, 544 nm, 584 nm and 620 nm, which attributes to the characteristics transition of ^5^*D*_4_→^7^*F*_6_, ^5^*D*_4_→^7^*F*_5_, ^5^*D*_4_→^7^*F*_4_ and ^5^*D*_4_→^7^*F*_3_, respectively (Figure S4).


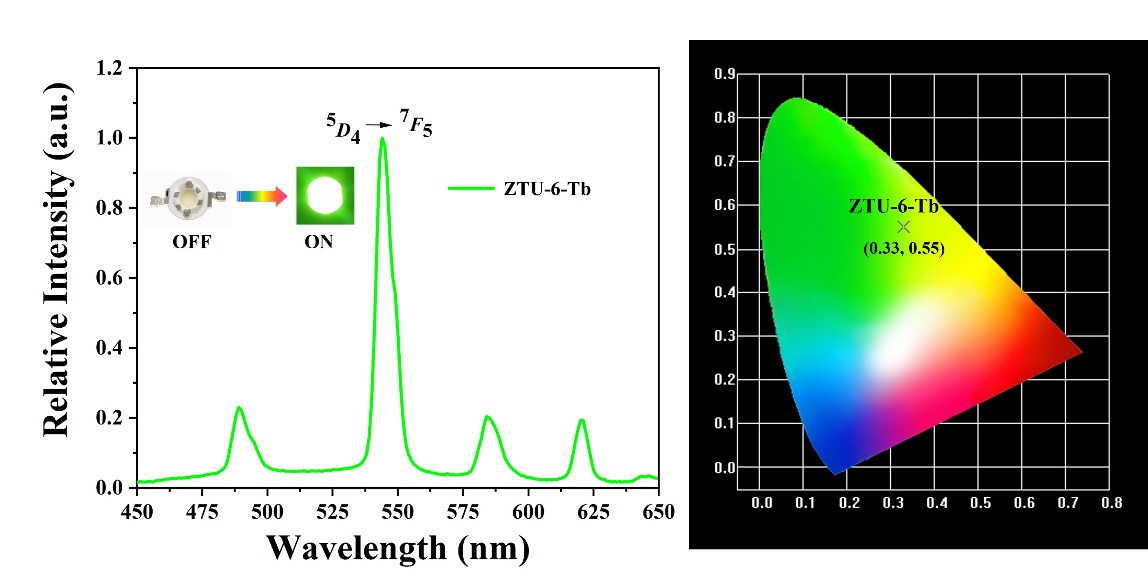


Figure S4 Emission spectroscopy and the CIE of the LED device with the **ZTU-6-Tb**.

Furthermore, the quantum yields and fluorescence lifetime of **ZTU-6-Eu** and **ZTU-6-Tb** were 86.87% and 85.62%, and 1.35 ms and 1.18 ms, respectively. The crystal materials of **ZTU-6-Eu** and **ZTU-6-Tb** were fully mixed with AB silica gel in a 1:1 mass ratio and then encapsulated on a commercial 365 nm UV LED chip to obtain red- and green-light LED devices. At 20 mA, the CIE coordinates are (0.63, 0.35) and (0.33, 0.55), and their CCT are 1110 and 5528, respectively.
